# Carbohydrate supplementation for endurance exercise in the heat: a systematic review with practical recommendations

**DOI:** 10.1080/15502783.2026.2669307

**Published:** 2026-05-09

**Authors:** Adriana Salame, Danny Brown, Krystel Oueijan, Deaglan McCullough

**Affiliations:** a Carnegie School of Sport, Leeds Beckett University, Leeds, UK; b Faculty of Agriculture and Food Sciences, American University of Beirut, Beirut, Lebanon

**Keywords:** Carbohydrate, endurance, gastrointestinal distress, heat, performance, thermoregulation

## Abstract

**Background and Objectives:**

Endurance exercise performance is impaired by heat stress. Although the mechanisms are not yet fully understood, heat-induced increases in glycogenolysis may play a role. Therefore, carbohydrate supplementation recommendations may differ for endurance exercise in hot environments. This systematic review aims to investigate the efficacy of carbohydrate supplementation for endurance performance in hot environments.

**Methods:**

Electronic databases, including PubMed, the Cochrane Library, MEDLINE, and SPORTDiscus were searched through to April 14, 2026 for studies evaluating endurance performance outcomes under heat stress in response to carbohydrate supplementation. Studies were included if the participants were healthy, aged 18–65 years, and at least recreationally active. A hot environment was defined as an ambient temperature >23 °C. Studies were required to involve an endurance-based and continuous exercise protocol lasting >30 min and a direct performance measure. This cutoff was selected based on evidence indicating glycogen stress at ~40 min under heat stress. In total, 3151 records were identified, and nine randomized, crossover studies met the inclusion criteria. Risk of bias was assessed using the Cochrane Collaboration's tool for assessing risk of bias. Primary outcomes (endurance performance and carbohydrate oxidation rates) and secondary outcomes (including gastrointestinal symptoms, thermoregulation, and hydration markers) were extracted. Mean carbohydrate intake rates were calculated, and a narrative synthesis was performed.

**Results:**

Mean carbohydrate ingestion rates ranged between 14 and 140 g·h^−1^, and the exercise trial duration ranged between ~50 and 152 min. Carbohydrate supplementation resulted in equivocal benefits to endurance performance: five studies found significant (*p* < 0.05) positive effects for carbohydrates (increases of 13.4%–19.3% in time to exhaustion and 3.3%–12.7% in time trial), and four studies found no significant effects. Although nonsignificant, three studies reported average improvements of 6.7%–11.6%, which may be meaningful in elite athletes. Most studies reported no differences in the respiratory exchange ratio between trials, indicating a preferential reliance on glycogen, which is in line with the literature. Carbohydrate ingestion during exercise in the heat did not influence markers of hydration, thermoregulation, or fatigue compared with placebo. Given that prior research shows gastrointestinal symptoms is common in endurance events in hot environments, the absence of symptom investigations in seven out of nine studies reviewed has important implications for interpreting findings. Overall, the results suggest that carbohydrate intake may not reduce glycogen breakdown during endurance performance in heat. Future research should help to better understand the underlying reasons, including any moderating effects of gastrointestinal distress.

**Conclusions:**

Carbohydrate intake during endurance exercise in the heat does not consistently improve exercise performance. Athletes may focus on maintaining hydration during exercise and practice gut-training, based on the wider literature. Future studies should fill the gaps in research, namely, the measurement of gastrointestinal symptoms, the mechanisms of exogenous carbohydrate use in heat, and different modes of exercise, including running, to enable the development of robust, evidence-based recommendations.

## 1. Background

Muscle and liver glycogen critically determine fatigue during prolonged exercise [1]. While research conflicts on whether carbohydrate ingestion during exercise spares muscle glycogen, it consistently demonstrates reduced liver glycogenolysis [2]. Widespread consensus exists on the ergogenic role of carbohydrates for endurance performance, with recommendations on the optimal amount, timing, and type for varying exercise intensities and durations in thermoneutral conditions [3]. Carbohydrate supplementation is common in endurance events, largely demonstrating performance improvements; however, outcomes vary with different supplementation and exercise protocols [4].

Glucose oxidation rate peaks at ~1 g·min^−1^ and coingesting fructose markedly increases total carbohydrate oxidation, elucidating the rate-limiting transport capacity of sodium-dependent glucose transporter 1 [5]. Accordingly, multiple transportable carbohydrates are recommended with ingestions >60 g·h^−1^, to minimize nutrient malabsorption, intestinal osmotic gradient shifts, gastrointestinal distress, and performance improvements [6]. Gastrointestinal disturbances are prevalent among endurance athletes, and overdosing the gut impairs exercise performance [7]. Numerous factors, including exercise intensity and duration, environmental conditions, individual predispositions, sex, age, and training status, may moderate the incidence and severity of gastrointestinal complaints and their impact on endurance performance [8,9].

Data from six World Athletics Gold-Labelled Marathon races determined that the environmental factor having the largest impact on exercise performance was the heat; at ~26 °C, average performance decreased by ~15.5% compared with an ideal value of ~6.5 °C [10]. Similarly, the cycling time-to-exhaustion at 70% VO_2max_ is reported to decrease by ~30 min between trials performed at 21 °C (81.2 min) and 31 °C (51.6 min), respectively [11]. Hence, while not all endurance events reveal a linear relationship between exercise performance and hot temperatures, exercise capacity is evidently impaired in hotter environments [12].

Understanding the underlying physiological mechanisms is fundamental to develop targeted interventions. Alongside the thermoregulatory strain resulting from metabolic heat production during exercise, hot environments also exacerbate thermoregulatory stress [13]. Accordingly, the body relies on cutaneous vasodilation and sweat loss to modulate heat efflux [14]. Although initial hypotheses suggested that circulatory failure impaired exercise performance, studies later revealed that thermoregulatory mechanisms did not occur at the expense of muscle perfusion [15,16]. Rather, progressive dehydration-related hyperthermia, defined as a core body temperature >40 °C, frequently coincided with exhaustion and is widely thought to involve central nervous system (CNS) inhibition [17].

In skeletal muscle, dehydration-induced hypoperfusion and hyperthermia increase glycogenolysis, the metabolic rate, and lactate production through sympathoadrenal activation [18,19]. Notably, glycogenolysis appears to be attenuated with heat acclimatization [20]. By similar mechanisms, splanchnic-hepatic hypoxia markedly increases liver glucose output during heat stress [21]. Beyond exercise-induced gastrointestinal disturbances, cardiovascular strain further damages the gastrointestinal barrier and impairs gastric emptying, perpetuating a cycle of heat stress-associated dehydration, tissue hypoperfusion and impaired fluid, electrolyte, and energy replacement [9]. While the mechanisms underlying fatigue development remain poorly understood, multifactorial physiological processes are implicated, including hyperthermia-induced central fatigue, peripheral effects on muscle activation, dehydration, and energy depletion [22–24].

Carbohydrate ingestion during exercise and heat stress may primarily delay fatigue by counteracting glycogen depletion. While research has robustly demonstrated that hepatic glucose output is suppressed by carbohydrate supplementation under thermoneutral conditions, heat glycogenolysis may not be attenuated [25]. In contrast, muscle glycogenolysis decreases with carbohydrate ingestion, which is consistent with reduced muscle fatigue under hot conditions [26]. However, exercise exhaustion under heat stress strongly correlates with hyperthermia rather than glycogen depletion, and carbohydrate ingestion may be associated with a heightened core temperature [27,28]. Nonetheless, through central effects, carbohydrate intake may attenuate early fatigue onset and subsequently improve endurance performance [29].

Carbohydrate beverage composition may also critically influence hydration [30]. While rapid gastric emptying and increased plasma volume are reported with hypotonic carbohydrate-electrolyte solutions (2%–3%), greater carbohydrate concentrations (~6%) may improve fluid retention through insulin-mediated renal sodium reabsorption [31,32]. However, most studies have been conducted largely at rest and may not fully account for exercise-induced delayed gastric emptying, which is exacerbated in the heat [9]. This diminished fluid delivery may exacerbate dehydration, nutrient malabsorption, and gastrointestinal symptoms [33,34].

Carbohydrate supplementation during exercise in hot environments may spare endogenous glycogen and modify critical aspects of endurance performance, including fatigue, gastrointestinal disturbances, hydration, and, potentially, thermoregulation. Recommendations for athletes performing heat are centered around hydration [35], and gastrointestinal management strategies are focused mostly on supplements and pre-event dietary restrictions [36]. To date, no systematic review has been performed on carbohydrate supplementation during endurance exercise in the heat. Thus, this systematic review aims to investigate the efficacy of carbohydrate supplementation for endurance performance in hot environments.

## 2. Methods

This systematic review was completed in accordance with the PRISMA (Preferred Reporting Items for Systematic Review and Meta-analyses) guidelines [37]. The protocol of this work was predefined and registered in PROSPERO, registration number CRD42024579652.

### 2.1. Search strategy

A comprehensive literature search was performed in the electronic databases of PubMed, MEDLINE, SPORTDiscus, and the Cochrane Library, through April 14 2026. The search strategy was completed using the following combination of keywords: (“carbohydrate supplement*” OR carbohydrate* OR “carbohydrate intake” OR “carbohydrate solution” OR “carbohydrate drink” OR “carbohydrate ingestion” OR glucose OR fructose OR sucrose OR dextrose OR maltodextrin OR sugar* OR carbohydrate-electrolyte OR carbohydrate-protein OR “sports drink” OR isotonic OR hypertonic OR hypotonic) AND (endurance OR running OR run OR cycl* OR exercise OR aerobic OR training) AND (performance OR “time trial*” OR capacity OR speed OR “power output” OR fatigue OR glycogen) AND (heat OR hot OR hot-humid OR warm OR warm-humid). Reference lists of eligible studies and review articles were also searched. Publication date and language restrictions were not applied.

### 2.2. Eligibility criteria

Studies were included if the participants were healthy, at least recreationally active [38]; aged 18–65 years; nonsmokers; not pregnant; and without any history of diabetes, gastrointestinal, inflammatory, metabolic, cardiovascular, neurological, or psychological disease(s). These criteria were selected to eliminate any confounding effects on exercise outcomes. Gray literature was included to minimize potential publication bias. Reference lists of relevant studies were reviewed for potentially eligible studies.

All studies were required to contain a carbohydrate-based supplement ingested during exercise as their intervention(s) and an equivalent comparative or control trial differing in carbohydrate content only. Carbohydrate interventions combining other ingredients (e.g. protein, caffeine) were included.

Studies were required to involve an endurance-based and continuous exercise protocol and a direct endurance performance measure, including a time trial and time to exhaustion. Protocols with brief interruptions of <5 min solely for data collection purposes were considered eligible. Exercise duration was required to last >30 min to account for muscle glycogen stress, which was originally reported after ~40 min of exercise in the heat [39,40]. Heat exposure was defined as either exposure to a true hot environment via season or geographical location (e.g. summertime, tropical country) or simulated heat exposure (e.g. heat chamber). A hot environment was defined as an ambient temperature >23 °C [41,42].

An initial assessment of studies for inclusion was carried out by two authors (AS and DB) until February 2023, followed by a second assessment of studies up until September 1 2024 (AS and KO) and a third assessment of studies through April 14 2026 (AS and DM). Disagreement regarding inclusion was discussed with the DM or DB. Studies that could potentially be included based on their title or abstract were retrieved in full text and evaluated against the eligibility criteria.

### 2.3. Data extraction

Data were extracted by one author (AS) and screened by two authors (DM or DB) into an Excel spreadsheet, which included the characteristics of studies valid for review. Additional data were collected on the study design, ambient temperature and humidity, participant characteristics, acclimatization and training status, gastrointestinal complaints, core temperature, hydration markers, substrate utilization, secondary performance measures, and subjective measures. When a study performed multiple consecutive exercise trials for each condition, data for the first trial were extracted only. Where values were only presented in figure form, the figure was digitized using graph digitizer software (DigitizeIt, Germany), and the mean and standard deviation were manually measured to the scale provided on the figure.

### 2.4. Risk of bias assessment

Risk of bias in the included studies was assessed by two authors (AS and DM or DB) using the Cochrane Risk of Bias (RoB) Tool V.2. [43], encompassing five domains: the randomization process, deviation from intended interventions, missing outcome data, measurement of the outcome, and selection of the reported results. Each potential source of bias was graded as low risk, some concern or high risk of bias. Disagreements were settled by consensus among the authors or through consultation with a third reviewer (DM or DB).

### 2.5. Data synthesis

Primary outcome findings of individual studies and their statistical significance were presented, and absolute (carbohydrate−placebo) and percentage $\left(\frac{\mathrm{carbohydrate}-\mathrm{placebo}}{\mathrm{placebo}}\right)\;x\;100$ differences between groups were calculated. Mean carbohydrate intake rate was calculated in g·h^−1^ and presented alongside the supplementation protocols. Exact *p* values were reported per the included studies, and statistical significance was considered at *p* < 0.05. Both the study characteristics and a summary of the findings were tabulated, and the results were synthesized narratively.

## 3. Results

### 3.1. Literature search and study characteristics

**Figure 1.**
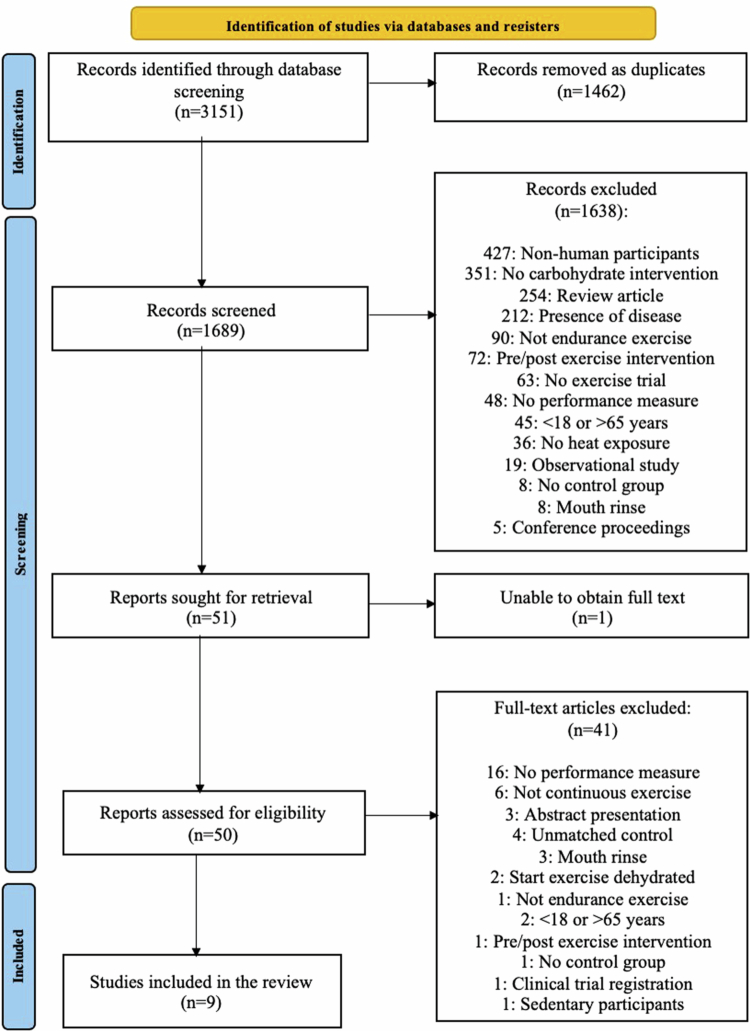
Preferred reporting items for systematic reviews and meta-analysis (PRISMA) study flow diagram.

The literature search (Figure 1) yielded 3151 records, and after the removal of duplicates, 1689 abstracts were screened. Fifty-one full texts were selected for review, and nine studies were included in the systematic review. At the time of inclusion, all studies were published in peer-reviewed scientific journals.

### 3.2. Participants

All studies were randomized, crossover trials (Table 1). Sample sizes ranged from 6 to 16 participants. Participants were mostly young (<30 years), trained with highly trained men, and two studies included both males and females. Four out of nine trials reported on acclimatization status, in which participants were nonacclimatized [29,44–46].

**Table 1.** Study characteristics.

Author	Study design	Environmental conditions (temperature, humidity)	Sample size	Sex	Age (mean ± SD)/ (range)	Maximal oxygen uptake (mL.kg^−1^.min^−1^) (mean ± SD)	Acclimatization status
Abbiss et al. [51]	Randomized double-blind crossover	32 °C, 55%	10	Male	27 ± 7	61.7 ± 5.0	No data
Carter et al. [29]	Randomized double-blind crossover	35 °C, 30%	8	Male	22.6 ± 1.1	59.5 ± 4.6	Nonacclimatized
Carter et al. [48]	Randomized blind crossover	35 °C, 30%	8	Male	24 ± 4	60.5 ± 6.7	No data
Che Jusoh et al. [47]	Randomized crossover	30 °C, 78%	8	Male	45 ± 4	65.0 ± 10.0	No data
Cureton et al. [50]	Randomized double-blind crossover	28.5 °C, 60%	16	Male	27.5 ± 7.0	60.5 ± 7.2	No data
Davis et al. [49]	Randomized double-blind crossover	~27 °C–28 °C, 67%	15	Male	20-31	63.0 ± 6.0	No data
Febbraio et al. [44]	Randomized blind crossover	33 °C, 20%–30%	HT1: 6 HT2: 6	Series 1: (HT1 or CT): 10 male, 2 female Series 2 (HT2): 6 male	HT1: 28.2 ± 1.2 HT2: 25.8 ± 1.9	HT1: 65.5 ± 3.9 HT2: 66.0 ± 1.9	Nonacclimatized
Flood et al. [45]	Randomized double-blind crossover	32 °C, 70%	14	7 Male, 7 Female	Male: 27 ± 8 Female: 23 ± 2	Male: 56.4 ± 7.6 Female: 54.3 ± 12.3	Nonacclimatized
Watson et al. [46]	Randomized crossover	30 °C, 60%	12	Male	21 ± 2	~52.4 (4.2 ± 0.5 L.min^−1^)	Nonacclimatized

CT = control trial in cool climate; HT1 = exercise trial 1 in the heat, HT2 = exercise trial 2 in the heat, SD = standard deviation, °C= celsius.

### 3.3. Setting and exercise mode

All nine studies were conducted in environmental chambers, with temperatures between 27 °C and 35 °C, and humidity between 20% and 78% (Table 1). All trials involved cycling.

### 3.4. Interventions and comparators

Most studies involved a single exercise protocol (*n* = 7), during which a single intervention (*n* = 4), two interventions (*n* = 2), and three interventions (*n* = 1), each were evaluated vs placebo. One study involved two exercise protocol series at different intensities, evaluating the same intervention, and another study involved two identical protocol series, which evaluated a total of three interventions, compared with placebo.

Most interventions (*n* = 8) involved carbohydrate solutions/beverages and were compared to flavor-matched, sweetened beverages (*n* = 7) or plain water (*n* = 1). One study involved solid food (sago) compared with nothing consumed (*n* = 1) [47].

Three studies investigated single transportable carbohydrate as glucose or maltodextrin, with a mean ingestion rate of 49.5–99.0 g·h^−1^[29,48,49], five studies explored multiple transportable carbohydrates, including sucrose, glucose/sucrose, glucose/sucrose/fructose, sucrose/dextrose, and maltodextrin/fructose drinks with average ingestion rates between 14 and 140 g·h^−1^ [44–46,50,51], and one study evaluated sago ingested at 62 g·h^−1^[47] (Table 2).

**Table 2.** Effects of carbohydrate supplementation during exercise lasting >30 min on endurance performance in the heat.

Author	Exercise and endurance performance description/duration	Pre-exercise meal carbohydrate content (g)	Carbohydrate type (study-dependent variable)	Mean carbohydrate intake rate (g·h^−1^)	Endurance performance outcome vs P (mean ± SD)	Absolute difference vs P	Mean percentage change vs P (%)	Significant performance effect vs *p*?
Abbiss et al. [51]	Cycling at 62% VO_2max_ for 90 min, followed by a 16-km time trial	78 (standardized diet in the morning of trial)	25% sucrose gel/solution (CHO); taste- and color-matched placebo (P) 0.48 g.kg^−1^ prewarm up, then 0.24 g kg^−1^ for 90 min	~75	CHO: 26.6 ± 1.4 min P: 27.5 ± 1.9 min	0.9 min faster	3.3% faster	Yes
Carter et al. [29]	Cycling at 60% VO_2max_ to exhaustion Cycling at 73% VO_2max_ to exhaustion	38 (5 min pre-exercise bolus)	6.4% sweetened maltodextrin drink (CHO); artificially colored and flavored placebo (P) 8 mL.kg^−1^ pre-exercise, then 3 mL.kg^−1^ every 15 min during exercise	60% VO_2max_ and 73% VO_2max_: ~58	60% VO_2max_: CHO: 145.6 ± 15.1 min P: 123.1 ± 13.4 min 73% VO_2max_: CHO: 60.6 ± 11.1 min P: 50.8 ± 7.5 min	60% VO_2max_: 22.5 min 73% VO_2max_: 9.8 min	60% VO_2max_: 18.3% 73% VO_2max_: 19.3%	Yes, both trials (60% VO_2max_: *p* = 0.028; 73% VO_2max_: *p* = 0.04)
Carter et al. [48]	Cycling at 55% PPO to exhaustion	39 (5 min pre-exercise bolus)	6.4% sweetened maltodextrin drink (SC); 6.4% nonsweet maltodextrin (NSC); water (P) 8 mL.kg^−1^ pre-exercise, then 3 mL.kg^−1^ every 15 min during exercise	~58	SC: 152.0 ± 18.3 min NSC: 145.1 ± 12.1 min P: 128.0 ± 14.1 min	SC: 24.0 min NSC: 17.1 min	SC: 18.8% NSC: 13.4%	Yes, both drinks
Che Jusoh et al. [47]	Cycling at 55% VO_2max_ for 45 min, followed by a 15-min time-trial	75.4 (2 h pretrial breakfast)	0.8 g.kg^−1^ Sago, divided into equal amounts at 0, 15, 30, and 45 min (CHO); nothing consumed (P)	~62	CHO: 219 ± 32 kJ P: 221 ± 33 kJ	−3 kJ	−0.9%	No
Cureton et al. [50]	Cycling for 120 min, alternating between 60% and 75% VO_2max_, followed by a 15-min time-trial	*n* = 12: 0; *n* = 4: small meal/CHO drink (≥3 h trial)	6% sucrose/dextrose-electrolyte drink (CES); artificially sweetened, colored placebo (P) 6 mL.kg^−1^ pre-exercise, then 3 mL.kg^−1^ at 15 min intervals for first 105 min	63.5 ± 6.0	CES: 190 ± 36 kJ P: 178 ± 31 kJ	12 kJ	6.7%	No
Davis et al. [49]	Cycling at 65% VO_2max_ for 1 h, followed by 270 pedal revolutions as fast as possible (~3 min)	0	12% glucose drink (HC); 6% glucose drink (MC); flavored water (P) 275 mL every 20 min, with first drink 10 min into exercise	HC: 99.0 MC: 49.5	HC: 149.8 ± 5.7 s MC: 151.3 ± 4.2 s P: 150.9 ± 3.7 s	HC: −1.1 s MC: 0.4 s	HC: −0.7% MC: 0.3%	No
Febbraio et al. [44]	HT1 and HT2: cycling at 70% VO_2max_ to exhaustion	0	HT1: 14% glucose/sucrose + glucose polymer drink (HCHO); 7% glucose/sucrose drink (NCHO); sweet placebo (P) HT2: 4.2% glucose/sucrose drink (LCHO); 7% glucose/sucrose drink (NCHO); sweet placebo + mannitol (P) 250 mL immediately pre-exercise, then every 15 min during exercise	14% drink: 140 7% drink: 70 4.2% drink: 42	HT1: HCHO: 74.0 min NCHO: 90.6 min P: 98.0 min HT2: LCHO: 97.5 min NCHO: 97.4 min P: 90.8 min	HT1: HCHO: −24.0 min NCHO: −7.4 min HT2: LCHO: 6.7 min NCHO: 6.6 min	HT1: HCHO: −24.5% NCHO: −7.6% HT2: LCHO: 7.4% NCHO: 7.3%	No, although time to exhaustion tended to be lower (*p* = 0.09) in HCHO in HT1 vs NCHO and P
Flood et al. [45]	Cycling at 45% VO_2max_ for 90 min, followed by a 15 min time-trial	Self-selected CHO-rich meal 3 h pretrial	16% maltodextrin/fructose drink (CHO); water (P) 250 mL in first 3 min, then ~143 mL every 15 min for 90 min	90	CHO: 169 kJ P: 150 kJ	19.1 kJ	12.7%	Yes (*p* = 0.005)
Watson et al. [46]	Cycling at 60% VO_2max_ to exhaustion	0	0% (P), 2%, 4% and 6% sucrose/glucose/fructose (ratio 50:25:25) fruit-flavored drinks 3 mL.kg^−1^ immediately pre-, then 1.5 mL.kg^−1^ every 10 min during exercise	2% drink: 14 4% drink: 29 6% drink: 43	0% drink (P): 94.5 ± 24.5 min 2% drink: 104.1 ± 20.1 min 4% drink: 105.5 ± 26.7 min 6% drink: 112.0 ± 28.7 min	2% drink: 9.6 min 4% drink: 11.0 min 6% drink: 17.5 min	2% drink: 10.2% 4% drink: 11.6% 6% drink: 18.5%	Yes, 6% drink (*p* = 0.045) No, 2% drink (*p* = 0.643) and 4% drink (*p* = 0.188)

CES = carbohydrate-electrolyte, CHO = carbohydrate, HC = high-carbohydrate, HCHO = high-carbohydrate, LCHO = low-carbohydrate, MC = medium-carbohydrate, NSC = nonsweet carbohydrate, NCHO = normal-carbohydrate, P = placebo, PPO = peak power output, SC = sweet carbohydrate, VO_2max _= maximal oxygen update.

### 3.5. Exercise protocols

Four studies involved time-to-exhaustion trials at intensities of 60%–73% VO_2max_ or 55% peak power output, and five studies involved steady-state cycling ranging between 45 and 120 min, immediately followed by a time trial between ~3 and ~28 min (Table 2).

### 3.6. Primary outcome measures

#### 3.6.1. Exercise performance

Five studies found significant benefits (*p* < 0.05) for carbohydrate supplementation on endurance performance in the heat, compared with placebo (Table 2). Two studies investigated 6.4% maltodextrin drinks (58 g·h^−1^), and described time-to-exhaustion improvements between 13.4% and 19.3% [29,48]. Another study employed a time-to-exhaustion trial and described a significant 18.5% endurance performance improvement with a 6% glucose/sucrose/fructose drink (43 g·h^−1^) and nonsignificant 11.6% and 10.2% improvements with 4% (29 g·h^−1^) and 2% (14 g·h^−1^) drinks, respectively [46]. One study reported a 3.3% performance improvement in a 16-km cycling time trial with a 25% sucrose solution (75 g·h^−1^) compared to placebo [51]. Finally, Flood et al. (2020) reported a 12.7% improvement in a 15-min cycling time trial with a 16% maltodextrin/fructose drink (90 g·h^−1^) compared with unflavored water [45].

The remaining four studies found no effect of carbohydrate supplementation on endurance performance in the heat (Table 2).

#### 3.6.2. Carbohydrate oxidation rates

In this review, eight studies, excluding Abbiss et al. (2008), used indirect calorimetry to compare carbohydrate oxidation rates between trials. Five studies reported no significant differences throughout exercise [29,46,47,49,50]. Febbraio et al. (1996) reported a significantly higher respiratory exchange ratio only for high-carbohydrate drinks (14%) compared with the normal-carbohydrate (7%) and placebo drinks in the first trial in a hot environment (HT1) [44]. Carter et al. (2005) showed a trend toward higher carbohydrate oxidation rates in both sweet and nonsweet carbohydrate drinks compared with water throughout exercise, and this trend was statistically significant (*p* < 0.05) at 45 min and 75 min [48]. Finally, Flood et al. (2020) reported a significantly higher carbohydrate oxidation rate throughout the exercise trial with the carbohydrate drink compared with water [45].

### 3.7. Secondary outcome measures

#### 3.7.1. Hydration and thermoregulatory mechanisms

In this review, seven studies investigated treatment effects on hydration status: five studies [29,45,47–49] measured body mass changes and blood hematocrit [52], Watson et al. (2012) [46] measured body mass change only and Febbraio et al. [44] assessed blood hematocrit. All studies reported similar fluid ingestion rates between trials. Two studies [44,47] found significant differences in hydration status between carbohydrates and placebo: Che Jusoh et al. [47] reported that sago porridge better maintained plasma volume and resulted in lower sweat losses than placebo, and Febbraio et al. [44] reported greater plasma volume loss with a 14% carbohydrate drink in the first trial at a hot temperature.

Eight studies, excluding Cureton et al. [50], investigated treatment effects on thermoregulation, mostly using rectal probe except two studies, which employed an ingested temperature pill [45,46]. Seven studies reported no significant differences in rectal/core temperature between carbohydrate and placebo trials (*p* > 0.05), and rectal/core temperatures, as depicted in the figures, peaked at ~38 °C–39 °C upon exercise termination, with a more rapid rise toward the end of performance. Abbiss et al. (2008) reported a significantly higher rectal temperature in the carbohydrate intervention group, compared with placebo (*p* < 0.05) [51].

#### 3.7.2. Rating of perceived exertion (RPE)

Seven studies investigated treatment effects on the RPE, with six studies [45–48,50,51] reporting no differences between carbohydrate and placebo. Conversely, Carter et al. [29] described a lower RPE trend in the 60% VO_2max_ time-to-exhaustion trial when ingesting the 6.4% sweetened maltodextrin drink compared with placebo.

#### 3.7.3. Gastrointestinal disturbances

Two studies investigated treatment effects on gastrointestinal symptoms. Davis et al. (1988) reported significantly greater nausea, fullness, and stomach upset for a 12% glucose drink (99 g·h^−1^), compared to a 6% glucose drink and placebo [49]. Flood et al. (2020) described greater fullness but no significant difference in gastrointestinal disturbances, with a 16% maltodextrin-fructose drink (90 g·h^−1^), compared to water [45].

### 3.8. Risk of bias

**Figure 2.**
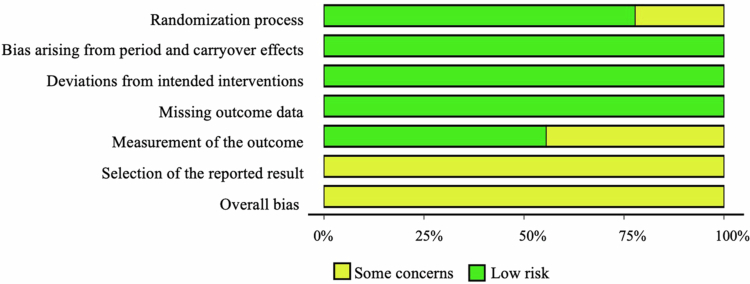
Risk of bias per domain for all studies using the RoB Tool V.2 [43].

The risk of bias assessment of all ten eligible studies was evaluated using the RoB 2 tool and is presented in Figure 2 and Table S1. The overall risk of bias across all crossover trials (*n* = 9) raised some concerns. Selection of the reported results, measurement of the outcome, and randomization were the domains that raised concerns in some of the studies.

## 4. Discussion

This systematic review aimed to investigate the efficacy of carbohydrate supplementation for endurance performance in hot environments. Five studies found significant (*p* < 0.05) improvements ranging from 13.4% to 19.3% improved time to exhaustion and 3.3%–12.7% in time-trial, while four studies found no effect (Table 2). Eight studies assessed carbohydrate oxidation rates via indirect calorimetry; three studies found significant increases, with 14% and 16% multitransportable carbohydrate drinks and a 6.4% maltodextrin drink [44,45,48]. Among the six studies investigating hydration status, one reported significant improvement with sago porridge, and another one, deterioration with a 14% carbohydrate beverage [44,47]. All the studies, except one [50], investigated thermoregulatory markers, and only Abbiss et al. (2008) reported a higher core temperature with carbohydrate intervention [51]. Six out of seven studies measuring RPE showed no difference between trials; one study reported a trend towards a lower RPE in the 60% VO_2max_ time-to-exhaustion trial [29]. Two studies have assessed gastrointestinal disturbances: Davis et al. (1998) demonstrated significantly greater gastrointestinal upset with a 12% glucose drink (99 g·h^−1^) compared with a 6% drink and water [49], and Flood et al. (2020) reported greater fullness (*p* < 0.05) with the maltodextrin-fructose drink vs water [45].

### 4.1. Primary outcomes: endurance performance and glycogen depletion

In this review, the ergogenic effects of carbohydrates on endurance performance in the heat, compared with flavor-matched placebos (*n* = 7), water (*n* = 1), or nothing consumed in a food-based trial (*n* = 1), are equivocal (Table 2). Contrary to research under thermoneutral conditions, five out of eight trials found no significant differences in carbohydrate oxidation, potentially indicating an antecedent reliance on glycogen stores under heat stress. However, no studies used isotope-labeled glucose tracers to differentiate between tissue and exogenous carbohydrate utilization [25]. Recent research also indicates that exercise intensity may regulate the effects of heat stress on substrate metabolism. In athletes of lower training status (VO_2max_ = 56.5 mL.kg^−1^.min^−1^) and under similar environmental conditions (~35 °C; 60% humidity); however, heat stress-induced changes in carbohydrate oxidation were reported to be unlikely below exercise intensities of 81 ± 8% VO_2max_[53]. As such, the studies on athletes included in this review (Table 1) undertaking exercise at 45%–70% VO_2max_ may not have elicited an intensity critically affecting substrate metabolism. Alternatively, large interindividual discrepancies in maximal fat oxidation may further confound the impact of heat stress on carbohydrate oxidation, notably at these exercise intensities [54,55]. Importantly, lacking data on the acclimatization status of participants (Table 1) has critical implications for interpreting findings, given that acclimatization reduces heat stress-associated glycogenolysis and its influence on endurance performance [56].

In the absence of within-subject variability data, lower coefficients of variability (~1%–2%) in endurance and mostly high-performance athletes (Table 1) merit critical interpretation of the findings with respect to real-life significance, where performance enhancements of 0.5%–1% may be considered worthwhile in competitive endurance events [57]. Notably, Cureton et al. (2007), Febbraio et al. (1996), and Watson et al. (2012) highlighted average improvements of 6.7%–11.6% in endurance performance, which may be deemed worthwhile in athletes with mean VO_2max_ values of 60.5, 66.0, and 52.4 mL.kg^−1^.min^−1^, respectively, despite not being statistically significant [44,46,50]. Interestingly, Cureton et al. (2007) described a higher carbohydrate oxidation rate and 23% endurance performance improvement with carbohydrate + caffeine compared with a placebo [50]. Whether the ergogenic effects of caffeine were related to the suppression of muscle glycogenolysis and increased exogenous carbohydrate oxidation or, as widely accepted, are independently attributable to CNS stimulation merits further investigation [58–60]. Moreover, in Febbraio et al. (1996), ingesting a 14% drink (~140 g·h^−1^), which significantly exceeds consensus recommendations of 30–60 g·h^−1^ for exercise 1–2.5 h [3], increased carbohydrate oxidation and resulted in a 32% (*p* = 0.09) decrease in endurance performance [44]. These findings align with those of King et al. (2019), where overdosing intestinal transport increases muscle glycogen reliance and impairs endurance performance[7]. While the underlying mechanisms are unclear, both studies and current evidence critically highlight the role of the gut in mediating the benefits of carbohydrate supplementation for endurance performance, notably in hot environments [33]. Altogether, while the possibility of muscle glycogen sparing with carbohydrate ingestion in the heat cannot be eliminated, the overall impact on endurance performance remains unclear, emphasizing that the complex interplay of multiple physiological processes during exercise under challenging environmental conditions [25,26].

Study protocol variations may also help to explain differences in performance outcomes. The inclusion of a pre-exercise meal in four studies (Table 2), which found mixed effects for the nutritional interventions, may not only moderate the effectiveness of carbohydrates in fasted athletes but also better represent the real-world competition context [61]. While all the studies were conducted in an environmental chamber, which facilitates data collection, research importantly describes differences in the benefits of carbohydrate supplementation on endurance performance during field-based trials, demonstrating no effects or deterioration of endurance performance [62–64]. Despite the underlying reasons being unclear, consideration with respect to the applicability of results within the real-world context is critical. Alternatively, four studies involved a time-to-exhaustion trial while the other five included a time trial (Table 2). Time-to-exhaustion tests have been critiqued for their low reproducibility [65]. Indeed, Laursen et al. (2007) reported a mean coefficient of variation of 15.1% [9.8%–33.2%] for time-to-exhaustion trials conducted at each participant’s 5 km time-trial pace, whereas actual 5 km time trials demonstrated ~2%–3% mean coefficients of variation [66]. Interestingly, three studies using a time-trial protocol found no effect of carbohydrate supplementation on endurance performance in heat. Nevertheless, whereas time trials may prove superior in their more accurate detection of the smallest worthwhile changes in performance, the former may not uniquely explain the disparity of results. Nonetheless, two studies involved durations that lasted around 1 h [47,49]. While Febbraio et al. (1994) described glycogen stress within 30 min at 40 °C with 20% humidity, such durations, at less extreme environmental conditions (Table 1), may not have critically elicited glycogen stress. In addition, one of the studies provided a substantial pre-exercise meal [47], which may have reduced the potential benefits of carbohydrate supplementation. Finally, most studies (*n* = 7) used a fan for air circulation, while Febbraio et al. (1994) did not mention the use of a fan [39], potentially incurring different thermoregulatory strains. Still, the use of fans is consistent with real-world context [67] and standard laboratory protocols and reflects an important dimension of ecological validity captured in the review. Overall, these methodological differences in exercise duration, pre-exercise meals, and thermoregulatory strain may have contributed to the variability in outcomes and should be considered in the interpretation of the overall evidence.

### 4.2. Secondary outcomes

#### 4.2.1. Gastrointestinal disturbances

Only two studies evaluated gastrointestinal symptoms [45,49]. These findings align with the consensus guidance recommending 6%–8% single-carbohydrate concentrations and greater tolerable doses with multiple transportable carbohydrates during endurance exercise under thermoneutral conditions [68]. However, gastrointestinal symptoms are prominently exacerbated in heat [69], notably at higher exercise intensities [70,71], and can not only impair endurance performance in heat [72] but also intestinal permeability, which may contribute to the development of heat illness [73]. In fact, in Febbraio et al. (1996), a 32% decrease in endurance performance with a 14% carbohydrate drink, whereby no differences were observed in hydration or core temperature, may likely be explained by gut discomfort [44].

Lacking data on gastrointestinal symptoms limits the understanding of their influence on exercise performance and the optimal carbohydrate dose and type. Given that carbohydrate ingestion in heat affects markers of gastrointestinal distress and increases intestinal epithelial injury and that no studies have yet compared nutrient malabsorption at different ambient temperatures, it can only be speculated that lower concentrations of multiple transportable carbohydrates may be needed under hot conditions to maintain gastrointestinal health and function [6,69]. Additionally, all included studies involved a cycling protocol, with running often associated with a greater risk of gastrointestinal complaints [74]. Carbohydrate supplementation during running, particularly in the heat, is underrepresented in the current literature and warrants further research. Overall, whether the results of this review differ from those observed under thermoneutral conditions [75], partly due to gastrointestinal distress, merits further investigation. Given the benefits of gut training in moderating symptoms and improving nutrient absorption [33], future research may benefit from highlighting gut-training protocols.

#### 4.2.2. Hydration and thermoregulatory mechanisms

Hydration remains the critical nutritional factor affecting endurance performance in the heat, primarily through its role in moderating thermoregulatory efficiency [56]. Only two studies [44,47] found significant differences in hydration between interventions. Che Jusoh et al. (2016) reported that sago porridge better maintained plasma volume and resulted in lower sweat losses than did the placebo, reflecting the slower absorption of starch, which may improve fluid retention by reducing diuresis, possibly due to its small fiber content and lower insulin response [47,76,77]. Febbraio et al. (1996) reported greater plasma volume loss with a 14% carbohydrate drink, reflecting the wider literature on perturbations in hydration status with hypertonic solutions [44,78]. Altogether, results largely represent existing literature demonstrating similar gastric emptying rates between moderately concentrated carbohydrate beverages and water [79,80]. Thus, carbohydrates may not overwhelmingly influence hydration during endurance performance in the heat.

Thermoregulatory responses may not explain differences in performance outcomes. Across all studies, the core temperature peaked at ~39 °C upon exercise termination, reflecting pacing strategies that account for core temperature regulation [81]. While hyperthermia impairs endurance performance in heat [13], in this review, neither the maximal core temperatures of 39 °C, nor 30 °C–35 °C dry-bulb temperatures reflect such conditions of uncompensable heat stress. Conversely, under compensatory heat stress, carbohydrate availability is proposed to limit endurance performance [51]. Accordingly, the ergogenic effect of carbohydrates may have been moderated by other factors, including substrate oxidation, gastrointestinal effects, and/or central effects.

#### 4.2.3. Rating of perceived exertion

Seven studies investigated perceived exertion; six reported no differences between carbohydrates and placebo [45–48,50,51]. Carter et al. [29] described a trend of lower RPE in the 60% VO_2max_ time-to-exhaustion trial with a carbohydrate drink. Critically, these results must be interpreted in the context of endurance performance measures. The close relationship between pacing and RPE during time trials [82] predetermines performance time rather than RPE alone, which may vary between groups. Conversely, time-to-exhaustion trials, performed at the same power outputs, may better demonstrate differential RPE scores [83,84]. Under thermoneutral conditions, carbohydrate availability has consistently been shown to reduce perceived exertion and enhance endurance performance [85–88]; however, the findings of this review are in contrast. The greater psychobiological stress exerted by hot environments may increase perceived effort beyond the benefits of carbohydrate intake in normothermia [89–91]. Indeed, the reduced voluntary work rate observed in athletes performing in the heat underlines the extent of RPE escalation under thermoregulatory and cardiovascular strain [92]. Nonetheless, the limited evidence on mouth rinsing during exercise in hot environments may indicate a role for acclimatization in regulating central fatigue and is a suggested research avenue [93–95]. Interestingly, Cureton et al. (2007) reported a lower RPE in a carbohydrate + caffeine trial during a steady-state ride, highlighting the potential role of caffeine in mitigating perceived fatigue [50]. Altogether, it may be hypothesized that carbohydrate availability may not have consistently spared endogenous glycogen under heat stress, and/or its CNS effects were attenuated under such psychobiological stress and/or gastrointestinal complaints may have negatively impacted endurance performance [96,97].

### 4.3. Limitations

This systematic review has several limitations. First, the number of studies included was small due to methodological constraints and the limited number of published studies. Furthermore, a meta-analysis was not performed because of substantial clinical and methodological heterogeneity across studies, including differences in exercise protocols, carbohydrate dosing strategies, environmental conditions, and outcome measures. Second, given the absence of acclimatization data and some studies describing participants to be nonacclimatized, the findings may not be extrapolated to heat-acclimatized athletes [20]. Third, the majority of participants are young, well-trained males, who cannot accurately reflect other populations' needs [55,98]. Fourth, all the experiments were conducted in the laboratory, and seven out of nine used a fan for air circulation, incurring differential thermoregulatory stress compared with outdoor environments [67,99]. Fifth, all studies except one included a carbohydrate-based beverage as their intervention; therefore, this review may not provide information on the effects of solid or gel-based carbohydrate sources [100]. Sixth, overall bias for all studies was reported as “some concerns” due to a single question in one domain (5.1) related to not publishing a prespecified analysis plan that was finalized before unblinded outcome data; this is recommended to minimize selection bias in the future. Future research should prioritize diverse athlete populations, field-based performance, acclimatization control, and using various forms of carbohydrate supplementation to build on these findings.

## 5. Conclusions and practical recommendations

Carbohydrate supplementation during endurance exercise in the heat may not invariably improve exercise performance and has no overall effects on carbohydrate oxidation, hydration, thermoregulation, or perceived fatigue.

Importantly, the large heterogeneity in findings reflects real-world conditions, emphasizing an individualized athlete approach. Furthermore, it highlights the need for criticality in advising endurance athletes performing in the heat to supplement carbohydrates, given that supplement use must have a proven rationale. Practically, athletes should focus on maintaining hydration, adequately fueling pre-exercise, replenishing lost glycogen, and practicing gut training on the basis of the wider literature. Given rising global temperatures, and endurance events being frequently conducted outdoors, future studies in the heat are strongly merited, with an emphasis on the impact of carbohydrate strategies on gastrointestinal function (e.g. considerations for amount, timing, and type), acclimatization status, inclusion of diverse populations, modes of exercise (e.g., running), and study protocols (e.g., field-based studies).

## Supplementary Material

Supplementary Material Table_S1.docx
