# Supplementary material for: Carbohydrate supplementation for endurance exercise in the heat: a systematic review with practical recommendations
Source: J Int Soc Sports Nutr. 2026 May 9;23(1):2669307. doi: 10.1080/15502783.2026.2669307 (PMC13159610; doi:10.1080/15502783.2026.2669307)
Supplement: Supplementary Material — Table_S1.docx [file RSSN_A_2669307_SM1810.docx]

Table S1. Risk of bias per domain across studies using the Risk of Bias Tool 2

| **Author** | **Randomization process** | **Risk due to period and carryover effects** | **Deviations from intended interventions** | **Missing outcome data** | **Measurement of the outcome** | **Selection of the reported result** | **Overall bias** |
| --- | --- | --- | --- | --- | --- | --- | --- |
| Abbiss et al., 2008 | Low | Low | Low | Low | Low | Some concerns | Some concerns |
| Carter et al., 2003 | Low | Low | Low | Low | Low | Some concerns | Some concerns |
| Carter et al., 2005 | Some concerns | Low | Low | Low | Some concerns | Some concerns | Some concerns |
| Che Jusoh et al., 2016 | Some concerns | Low | Low | Low | Some concerns | Some concerns | Some concerns |
| Cureton et al., 2007 | Low | Low | Low | Low | Low | Some concerns | Some concerns |
| Davis et al., 1998 | Low | Low | Low | Low | Low | Some concerns | Some concerns |
| Febbraio et al., 1996 | Low | Low | Low | Low | Some concerns | Some concerns | Some concerns |
| Flood et al., 2020 | Low | Low | Low | Low | Low | Some concerns | Some concerns |
| Watson et al., 2012 | Low | Low | Low | Low | Some concerns | Some concerns | Some concerns |
